# IL-17B activated mesenchymal stem cells enhance proliferation and migration of gastric cancer cells

**DOI:** 10.18632/oncotarget.14835

**Published:** 2017-01-27

**Authors:** Qingli Bie, Bin Zhang, Caixia Sun, Xiaoyun Ji, Prince Amoah Barnie, Chen Qi, Jingjing Peng, Danyi Zhang, Dong Zheng, Zhaoliang Su, Shengjun Wang, Huaxi Xu

**Affiliations:** ^1^ Department of Immunology, School of Medicine, Jiangsu University, Zhenjiang, Jiangsu, China; ^2^ Department of Anesthesiology, The Affiliated Hospital of Jiangsu University, Zhenjiang, Jiangsu, China; ^3^ Department of Biomedical and Forensic Sciences, School of Biological Sciences, University of Cape Coast, Cape Coast, Ghana; ^4^ Key Laboratory of Laboratory Medicine of Jiangsu Province, School of Medicine, Jiangsu University, Zhenjiang, Jiangsu, China

**Keywords:** gastric cancer, IL-17B, mesenchymal stem cells (MSCs), stemness

## Abstract

Mesenchymal stem cells are important cells in tumor microenvironment. We have previously demonstrated that IL-17B/IL-17RB signal promoted progression of gastric cancer. In this study, we further explored the effect of IL-17B on mesenchymal stem cells in tumor microenvironment and its impact on the tumor progression. The results showed that IL-17B induced the expression of stemness-related genes Nanog, Sox2, and Oct4 in mesenchymal stem cells and enhanced its tumor-promoting effect. The supernatant from cultured mesenchymal stem cells after treating with exogenous rIL-17B promoted the proliferation and migration of MGC-803, therefor suggesting that rIL-17B might promote mesenchymal stem cells to produce soluble factors. In addition, rIL-17B also activated the NF-κΒ, STAT3, β-catenin pathway in mesenchymal stem cells. Our data revealed a new mechanism that IL-17B enhanced the progression of gastric cancer by activating mesenchymal stem cells.

## INTRODUCTION

Gastric cancer is one of the most common gastrointestinal malignancies, and the morbidity and mortality of patients with gastric cancer remain high. Tumor-associated stromal cells, as important components of the tumor microenvironment, perform an intricate cross-talk with tumor cells, thereby supplying appropriate signals that may promote tumor progression [1]. Many studies have demonstrated that mesenchymal stem cells (MSCs) are also present in tumor stroma, and play context-dependent roles in controlling tumor growth.

A substantial body of work have demonstrated the critical role of MSCs in immune regulation, and many different factors are believed to be involved, such as inducible nitric oxide synthase (iNOS), indoleamine 2,3-dioxygenase (IDO), interferon-γ (IFNγ), tumor necrosis factor-α (TNFα), CC-chemokine ligand 2 (CCL2), interleukin-10 (IL-10), interleukin-1 (IL-1), interleukin-6 (IL-6), interleukin-8 (IL-8), and prostaglandin E2 (PGE2) [2, 3]. Moreover, it is also evident that the immunomodulatory activity of MSCs is not innate, rather licensed by inflammatory cytokines, and is highly plastic in response to dynamic changes of inflammatory niche, including the concentration of inflammatory cytokines and the kinds of cytokines [4, 5]. Exposure to cytokines such as IFNγ, TNFα, IL-1β or IL-1α is known to enhance the immunosuppressive properties of MSCs [6]. However, interleukin 17A as one of the key inflammatory cytokines during immune responses, primarily produced by Th17 cells, is known to function as a MSCs growth factor [7]. And, IL-17 can in fact effectively synergize with IFNγ and TNFα in enhancing MSCs immunosuppression by promoting iNOS expression *in vivo* [8].

The IL-17 cytokine superfamily is composed of six structurally related cytokines including IL-17A, B, C, D, E, F [9]. IL-17B is not only involved in the immune response, but also related to development [10], fracture [11], and cancer [12, 13]. Our previous results found that IL-17RB was significantly increased in gastric cancer tissues than in paired non-cancerous tissues, the level of IL-17B in the serum of gastric cancer patients was also significantly higher than that in normal subjects, and the IL-17B/IL-17RB signal was pivotal for promoting the growth and migration of gastric cancer [14]. Wen-Hwa Lee and co-workers also clearly indicated the critical roles of IL-17B/IL-17RB signaling in cancer progression [12, 15].However, it is unclear whether IL-17B exhibits potential effects on MSC in the tumor microenvironment and contributes to tumor progression.

## RESULTS

### The supernatant of MSCs treated with rIL-17B promoted the proliferation and migration of MGC-803

**Figure 1:**
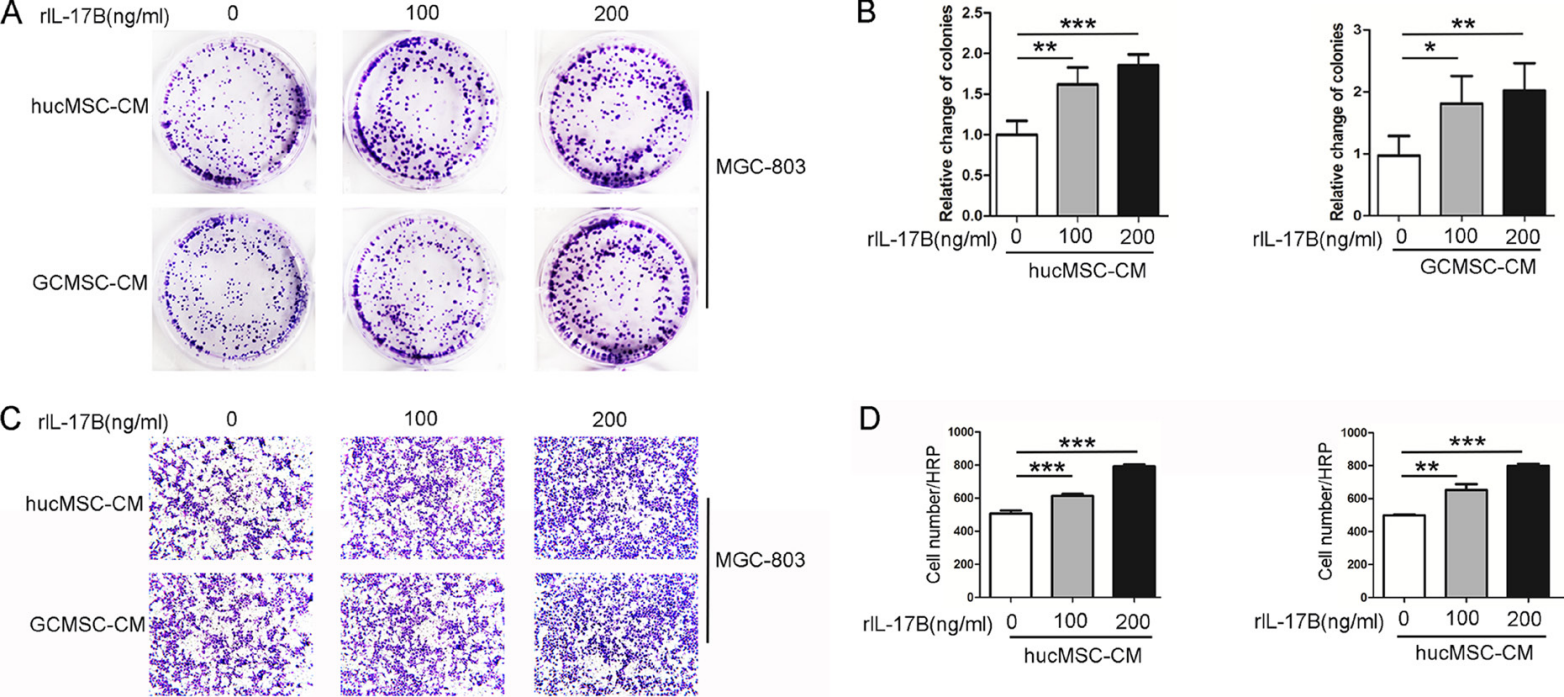
The supernatant of MSCs treated with rIL-17B promoted the proliferation and migration of MGC-803 (**A**) Representative images of cell colonies in MGC-803 cells stimulated with rIL-17B treated hucMSCs and GC-MSCs culture supernatants. (**B**) Relative change of colonies with > 100 cells, which were quantified in three random magnifications in MGC-803 cells stimulated with rIL-17B treated hucMSCs and GC-MSCs culture supernatants. (**C**) The migratory ability of MGC-803 cells stimulated with rIL-17B treated hucMSCs and GC-MSCs culture supernatants, which was evaluated by the Transwell^®^ migration assay. (**D**) The number of migrated MGC-803 cells was quantified. All samples were measured in triplicate, *p < 0.05, **p < 0.01, ***p < 0.001.

Tumor microenvironment is a very important factor in tumor development, in which MSCs’ role has attracted much attention. Human umbilical cord mesenchymal stem cells (HucMSCs) and gastric cancer-derived mesenchymal stem cells (GC-MSCs) were successfully isolated, cultured and characterized as previously described [16].In order to explore whether IL-17B has an effect on MSCs and further influences tumor progression, the hucMSCs and GC-MSCs were treated with rIL-17B for 48 hours, and washed with PBS for two times and incubated for another 48 hours, then this conditioned media was collected. Taking MGC-803 as a target, the colony forming assays and migration assays were performed. As shown in Figure 1A, 1B and 1C, the colony forming and migratory ability were significantly increased in a dose-dependent manner after MGC-803 was stimulated by supernatants, which were collected from hucMSCs and GC-MSCs treated with rIL-17B (Figure 1A–1D). These results suggested the inflammatory cytokines IL-17B could influence the paracrine activity of MSCs and further changed the biological behavior of gastric cancer cells.

### IL-17B prompted the proliferation and migration of MSCs

**Figure 2:**
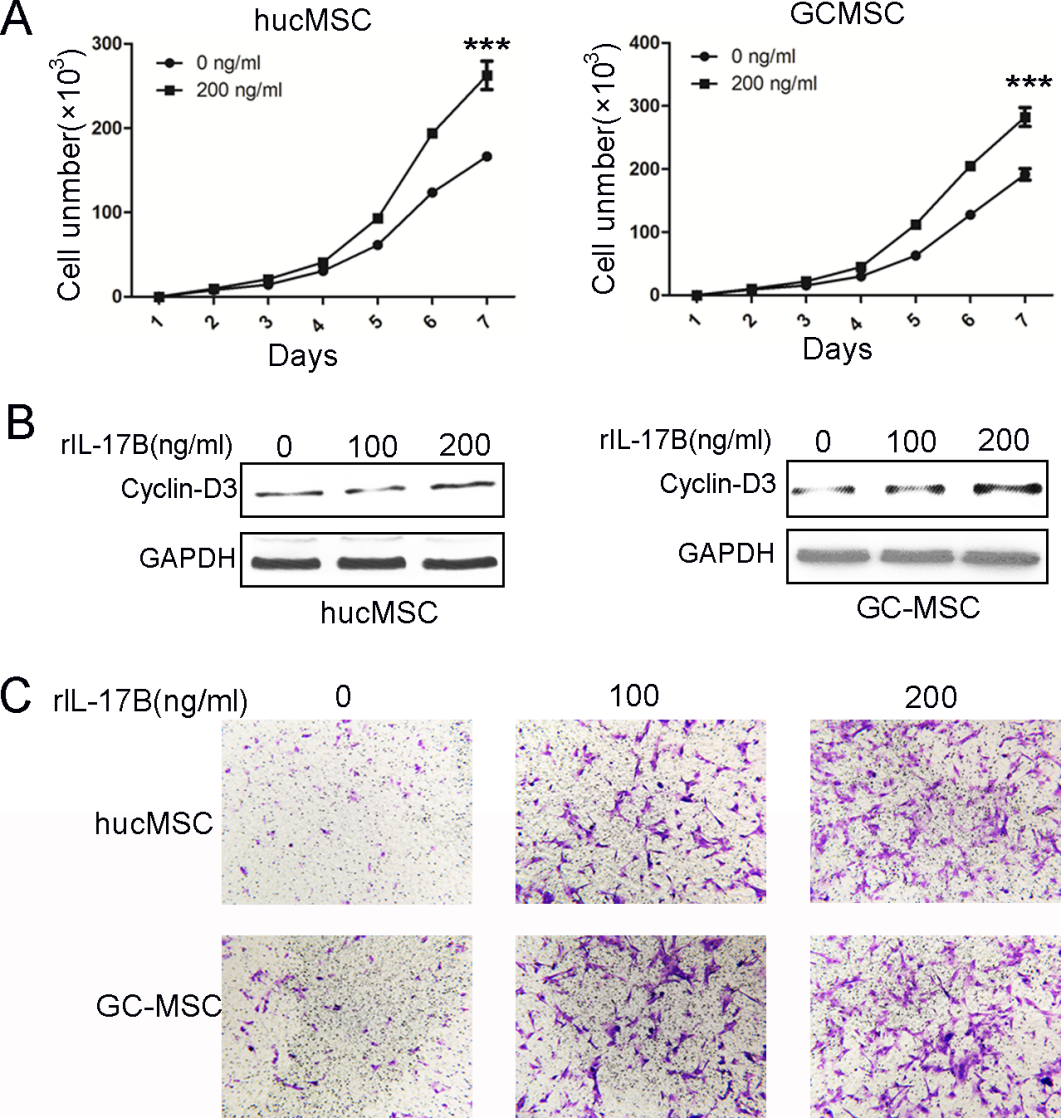
IL-17B prompted the proliferation and migration of MSCs (**A**) Cell-counting assay for the proliferating ability of hucMSCs and GC-MSCs treated with/without 200 ng/ml rIL-17B for 48 hours. (**B**) Western blot analyses of Cyclin-D3 protein expression in hucMSCs and GC-MSCs treated with/without 100 ng/ml and 200 ng/ml rIL-17B for 48 hours. (**C**) The migratory ability of hucMSCs and GC-MSCs treated with/without 100 ng/ml and 200 ng/ml rIL-17B for 12 hours was evaluated by the Transwell^®^ migration assay. All samples were measured in triplicate, ***p < 0.001.

The literatures have highlighted the role of IL-17A on murine BM-MSCs and human mesenchymal stem cells as a MSCs growth factor [7, 17]. To determine the effects of IL-17B on MSCs, we firstly evaluated the proliferation of hucMSCs and GC-MSCs treated with 200 ng/ml rIL-17B. The results of cell-counting assay showed that IL-17B significantly promoted the growth of hucMSCs and GC-MSCs (Figure 2A). Western blot results also showed that the expression of cyclin-D3 in hucMSCs and GC-MSCs treated with 100 or 200 ng/ml rIL-17B for 48 hours obviously increased in a dose-dependent manner (Figure 2B). These findings demonstrated that IL-17B promoted the proliferation of MSCs. MSCs migration to tumors is due to the soluble factors in tumor microenvironment including growth factors, chemokines and cytokines. In addition, we used transwell migration assay to analyze whether the migratory ability of MSCs are affected by IL-17B. The results showed that rIL-17B induced an enhanced migratory capacity in MSCs relative to the control in a dose-dependent manner (Figure 2C).

### IL-17B enhanced the stemness of MSC

**Figure 3:**
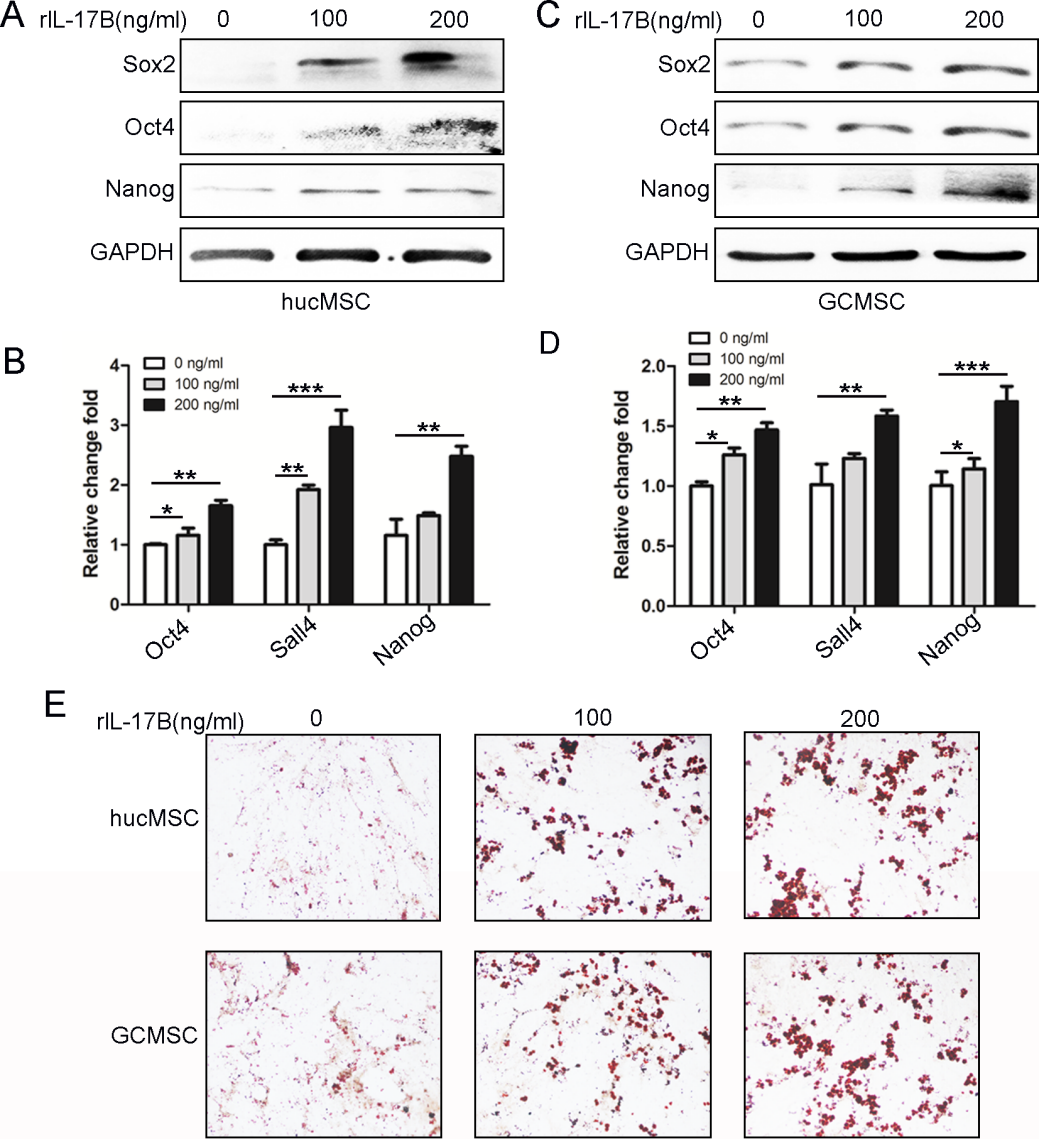
IL-17B up-regulated the stemness of MSC (**A** and **C**) Western blot analyses of Sox2, Oct4 and Nanog levels in hucMSCs (A) and GC-MSCs (C) treated with/without 100 ng/ml and 200 ng/ml rIL-17B for 48 hours. (**B** and **D)**. Quantitative analyses for relative mRNA levels of Oct4, Sall4 and Nanog in hucMSCs (B) and GC-MSCs (D) treated with/without 100 ng/ml and 200 ng/ml rIL-17B for 48 hours. (**E**) HucMSCs and GC-MSCs treated with corresponding concentrations of rIL-17B for 48 h, and then induced with adipogenic differentiation medium. Representative images show accumulation of lipid droplets after undergoing adipogenic differentiation. All samples were measured in triplicate, *p < 0.05, **p < 0.01, ***p < 0.001.

Recently, several investigations have brought to light the role of the interleukin-17 (IL-17) family of cytokines on influencing cancer stem-like cells [18–20]. Our previous data also showed that IL-17B/IL-17RB signaling promoted the expression of stemness-related genes Nanog, Sox2, and Oct4and enhanced MGC-803 cells efficiently to differentiate into adipocytes [14].To characterize the role of IL-17B on the stemness of MSC, we performed western blot to examine the expression of Sox2, Oct4, and Nanog in hucMSCs and GC-MSCs treated with different concentrations of rIL-17B. We found that the expression of these proteins was significantly increased in a dose-dependent manner (Figure 3A and 3C). A similar expression pattern at the mRNA level was also observed using qRT-PCR on hucMSCs and GC-MSCs (Figure 3B and 3D). We next determined the induced differentiation potential in hucMSCs and GC-MSCs treated with exogenous rIL-17B, and the results showed that hucMSCs and GC-MSCs could be efficiently induced to differentiate into adipocytes in the appropriate conditioned medium, and adipocytes increased in a dose-dependent manner (Figure 3E). These results suggested an important role of IL-17B in promoting hucMSCs and GC-MSCs stemness.

### IL-17B prompted the expression of soluble factors in MSC

**Figure 4:**
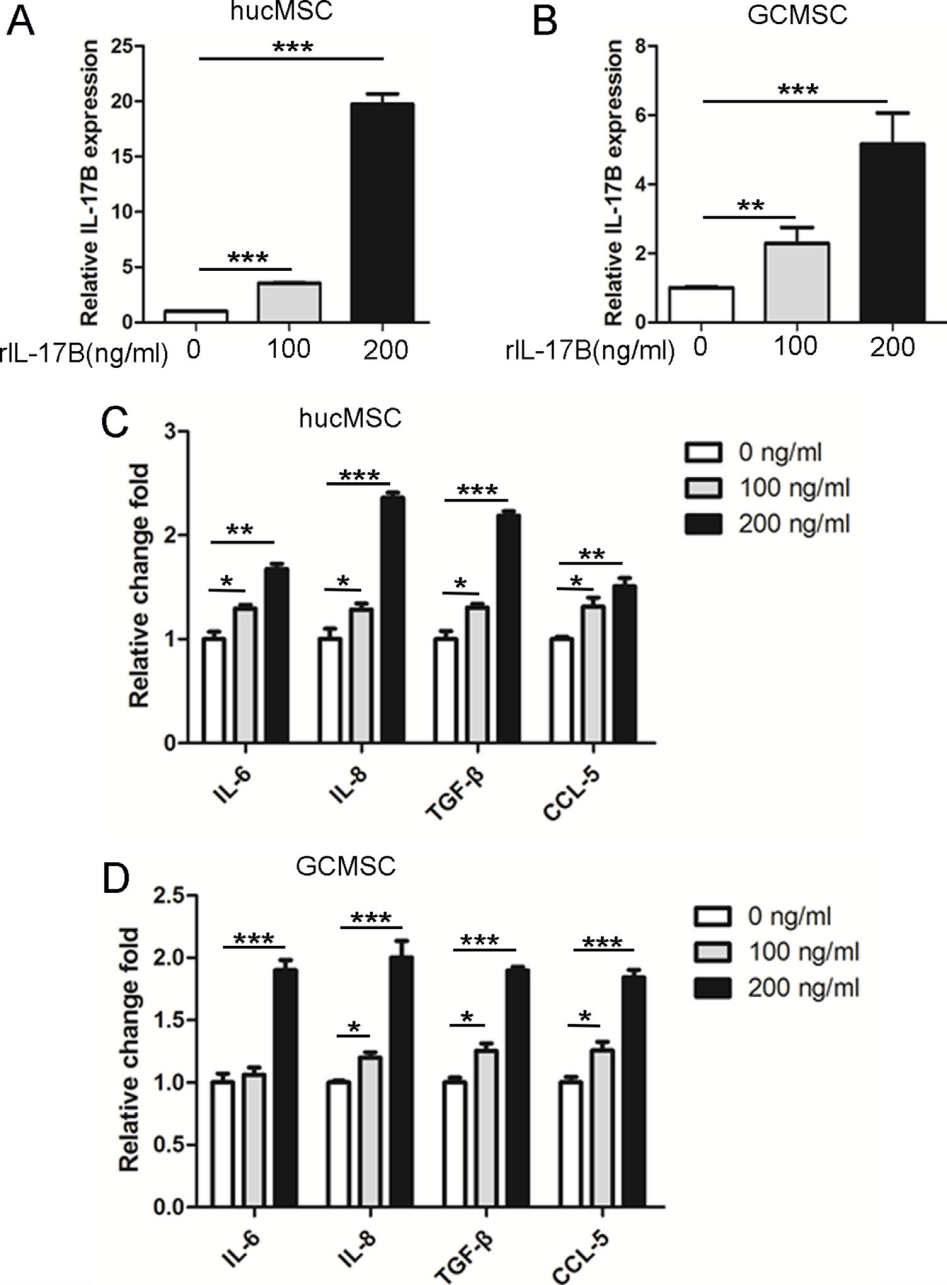
IL-17B prompted the expression of soluble factors in MSC (**A** and **B**) Quantitative analyses for relative mRNA levels of IL-17B in hucMSCs (A) and GC-MSCs(B) treated with/without 100 ng/ml and 200 ng/ml rIL-17B for 48 hours. (**C** and **D**) Quantitative analyses for relative mRNA levels of IL-6, IL-8, TGF-β and CCL-5 in hucMSCs (C) and GC-MSCs (D) treated with/without 100 ng/ml and 200 ng/ml rIL-17B for 48 hours. All samples were measured in triplicate, *p < 0.05, **p < 0.01, ***p < 0.001).

It is well established that MSCs exposed to pro-inflammatory cytokines express increased levels of TGF-β and IL-10 in order to exhibit more efficient immunomodulatory activity [21], as well as increased levels of IL-8 which are needed for angiogenesis [22]. Secreting cytokines, IL-6 and CXCL7 by MSCs can enhance CSCs proliferation to facilitate the tumor growth [23]. In order to investigate the effects of IL-17B on secreting of soluble factors in MSC, we cultured hucMSCs and GC-MSCs treated with 100 or 200 ng/ml of rIL-17B for 48 hours, then we performed qRT-PCR to detect the expression of IL-17B, IL-6, IL-8, TGF-β and CCL-5. The results showed that IL-17B not only enhanced the expression of IL-6, IL-8, TGF-β and CCL-5 of hucMSCs and GC-MSCs, but also enhanced IL-17B autocrine(Figure 4A–4D). These results demonstrated IL-17B could influence MSC to secret a variety of soluble factors.

### IL-17B activated NF-κΒ, STAT3 and β-catenin pathway in MSCs

**Figure 5:**
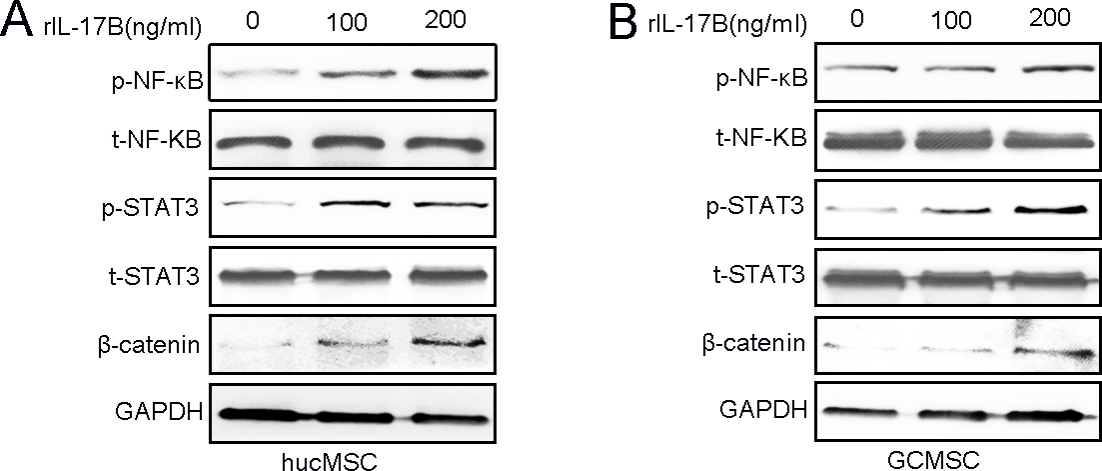
IL-17B activates the NF-κΒ, STAT3, β-catenin pathway of MSCs (**A** and **B**) The expressions of total and phosphorylated NF-κΒ, STAT3 and β-catenin were determined by western blot analyses of hucMSCs (A) and GC-MSCs (B) treated with corresponding concentrations of rIL-17B.

Wen-Hwa Lee and co-workers have reported that amplified IL-17B/IL-17RB signaling promoted breast tumorigenes through NF-kB-mediated anti-apoptotic pathway and activated downstream transcription factors NF- kB via ERK1/2 pathway to induce the expression of CCL20, CXCL1, TFF1, and IL-8 in both pancreatic cancer cells and the surrounding stroma [12, 13, 15]. Our previous results also indicated that the IL-17B/IL-17RB signal can activate the AKT/β-catenin pathway in gastric cancer [14].To elucidate how IL-17B promotes MSCs stemness, proliferation and migration, we analyzed the expression of proteins that could be activated by IL-17 family cytokines [24]. Western blot analysis showed that the expression of phosphorylated NF- kB, STAT3 andβ-catenin were significantly increased in a dose-dependent manner in hucMSCs and GC-MSCs after different concentrations of rIL-17B treatment (Figure 5A–5B).

## DISCUSSION

Mesenchymal stem cells are a subset of non-hematopoietic adult stem cells which originate from mesoderm and possess self-renew and multilineage differentiation ability [25]. Extensive investigations have shown that MSCs promote tumor growth and metastasis involving distinct aspects including angiogenesis, tumor cell survival, immunosuppressive microenvironment shape, as well as CSC maintenance and mesenchymal niche construction [26, 27]. The tumor-tropismproperties of MSCs make them ideal candidates used for tumor therapy as delivery vehicles of specific therapeutic genes [28]. It becomes increasing significant in understanding the role and fate of MSCs during tumor progression.

The roles of IL-17-family cytokines, especially IL-17A, in autoimmune diseases, allergic diseases and host defense against infection have gained extensive research [29–31]. Recently, several investigations have brought to light the role of the IL-17 cytokines family on influencing cancer stem-like cells [18–20]. Our previous data also showed that IL-17B/IL-17RB signaling promoted the expression of stemness-related genes Nanog, Sox2, and Oct4 and enhanced MGC-803 cells efficiently to differentiate into adipocytes [14].In addition, MSCs play an important role in the development of gastric cancers [32].We proposed a hypothesis that IL-17B may also indirectly promote gastric cancer progression by influencing MSCs.

IL-17A, as a MSCs growth factor, expanded human MSCs [7], and synergized with IFNγ and TNFα dramatically enhanced the immunosuppressive effect of MSCs by promoting iNOS expression [8]. IL-17A inhibits adipogenesis in hBM-MSCs and regulates pro-inflammatory responses by increasing cellular production of IL-6, IL-8 and prostaglandin E2 [33]. Some studies have indicated that IL-17B could induced TNFα and IL-1β expression from a monocytic cell line [34]. In this study, we demonstrated that IL-17B prompted the proliferation and migration of hucMSCs and GC-MSCs in a dose-dependent manner. In addition, IL-17B also enhanced the stemness and the secreting inflammation factors IL-6, IL-8, TGF-β and CCL-5 in hucMSCs and GC-MSCs. IL-17B increased the activation of NF- kB and STAT3, which induced the expression of inflammation related factors. Moreover, IL-17B up-regulated the stemness of MSCs, which enhanced the paracrine ability of MSCs, thence we detected some representative inflammation related factors to further support IL-17B could promote the stemness of MSCs. Considering Interleukin-8 (IL-8) could promote the proliferation and migration of cancer cells [35, 36], enhance tumor angiogenesis [37] and induce epithelial-mesenchymal transition of cancer cells [38]. And, studies have reported GC-MSCs prompt gastric cancer progression through secreting interleukin-8 [39]. We speculated IL-8 might play a relatively primary role in promoting the proliferation and migration of MGC-803. Beside those inflammation related factors in the supernatant, the activation of β-catenin induced by IL-17B in MSCs (Figure 5) also indicated that the secretion of Wnts may be another mechanism in this system. Recent study revealed that MSCs can secret exosomes delivered Wnt4 [40, 41], and exosomal Wnt4 could enhance gastric cancer cell stemness and tumorigenesis [42]. The various factors contained in the supernatant play a synergistic role in promoting tumors, therefore it is relatively difficult to resolve which soluble factor(s) might be responsible for the effects. In total, IL-17B also could indirectly promote gastric cancer progression by influencing the biological behavior and function of hucMSCs and GC-MSCs.

A wide range of tissue can express IL-17B, including the spinal cord, testis, stomach, small intestine, pancreas, prostate and ovary [34]. What pathological conditions can induce these tissues to express more IL-17B, especially chronic inflammationlike Helicobacter pylori (H. pylori) infection maybe induce increased expression of IL-17B in stomach. Again, elevated expression of IL-17B can promote the proliferation, migration and stemness of MSCs in circulation and tissues microenvironment, and further accelerates the tumor progression. We need further study to clarify these speculations in the future and to characterize the biological functions and the possible pathogenic roles of IL-17B in diseases.

## MATERIALS AND METHODS

### Cell culture and reagents

Gastric cancer cell lines MGC-803 were purchased from American Type Culture Collection (ATCC) and cultured in Dulbecco's modification of Eagle's medium (DMEM) containing 10% fetal bovine serum (Gibco, Grand Island, USA) at 37°C with 5% CO_2_ atmosphere. IL-17B were purchased from R&D corporation. Human umbilical cord mesenchymal stem cells (hucMSCs), gastric cancer-derived mesenchymal stem cells (GC-MSC) were isolated, cultured and characterized based on the literatures [16]. All experiment protocols were approved by the Ethics Committee of Jiangsu University.

### Colony-formation assay

Cells were harvested and seeded into six-well plate (800 cells/well) and incubated at 37°C in a 5% CO_2_ humidified incubator for 7 days. The medium was changed at 3-days interval. At the end of the incubation period, the cultures were fixed with 4% paraformaldehyde and stained with crystal violet.

### Transwell migration assay

Cells (1×10^5^/well) were plated into the top chamber and 10% FBS containing medium was placed into the bottom chamber. After incubation at 37°C in 5% CO_2_ for 12 h, the cells remaining at the upper surface of the membrane were removed with a cotton swab. The cells that migrated through the 8-μm sized pores and adhered to the lower surface of the membrane were fixed with 4% paraformaldehyde, stained with crystal violet and photographed.

### RNA extraction and quantitativereal-time PCR

Total RNA was extracted from cells using TRIzol reagent (Invitrogen, Carlsbad, CA, USA) according to the manufacturer's instructions. Two microgram aliquots of RNAs were synthesized according to the manufacturer's protocol (Vazyme R111-02, Nanjing, China). Quantitative real-time PCR (qRT-PCR) was conducted using the AceQ^®^ qPCR SYBR^®^ Green Master Mix (Vazyme Q111-02). β-actin was used as an internal control. The sequences of specific primers are listed in Table 1.

**Table 1:** Primer sequences

Target	Sequence (5′–3′)	Accession
Human IL-6	Fwd: TACATCCTCGACGGCATCTC	NM_000600.4
	Rev: AGCTCTGGCTTGTTCCTCAC	
Human IL-8	Fwd: GCTCTGTGTGAAGGTGCAGTTT	NM_000584.3
	Rev: TTCTGTGTTGGCGCAGTGT	
Human TGF-β	Fwd: CACACTGCAAGTGGACATC	NM_000660.5
	Rev: GCAGAAGTTGGCATGGTAG	
Human Nanog	Fwd: GCAATGGTGTGACGCAGAAG	NM_024865
	Rev: GCATGCAGGACTGCAGAGAT	
Human Oct4	Fwd: CAGAGTGGTGACGGAGACAG	NM_002701
	Rev: AGAAGGATGTGGTCCGAGTG	
Human CCL-5	Fwd: GGATTCCTGCAGAGGATCAA	NM_002985.2
	Rev: GTGGTGTCCGAGGAATATGG	
Human Sall4	Fwd: TCGATGGCCAACTTCCTTC	XM_011528919.1
	Rev: GAGCGGACTCACACTGGAGA	
Humanβ-actin	Fwd: TGGCACCCAGCACAATGAA	XM_005249820.1
	Rev: CTAAGTCATAGTCCGCCTAGAAGCA	

### Western blotting

HucMSC and GC-MSC were homogenized and lysed in RIPA buffer supplemented with proteinase inhibitors. Equal amounts of total protein were loaded and separated on 12% SDS-polyacrylamide gel. Following electrophoresis, the proteins were transferred to a PVDF (polyvinylidene difluoride) membrane (Millipore, USA), blocked in 5% (w/v) non-fat milk and incubated with the primary antibodies. Membranes were incubated with monoclonal antibody against GAPDH (CWBIO, CW0100),cyclin-D3 (Bioworld, BS6139), Nanog (SAB, 21423), Oct4 (Santa Cruz, SC-5279), Sox2 (Milipore, AB5603), p-STAT3 (Thy705) (Cell Signal Technology, 9145), t-STAT3 (Cell Signal Technology, 4904),β-catenin (Cell Signal Technology, 8480), p-NF-κB (Cell Signal Technology, 3033L), t-NF-κB (Cell Signal Technology, 8242s) at 4°C overnight. The membrane was washed with Tris-buffered saline/Tween (TBS/T) for three times and incubated with the secondary antibodies (Bioworld) at 37°C for 1 h. The signals were visualized by using Luminata crescendo western horseradish peroxidase (HRP) substrate (GE corporation, USA).

### Cell counting assay

In total, 5 × 10^3^ hucMSC and GC-MSC cells were plated in each well of 24-well plates for cell counting assay. The number of cells in each well was counted in triplicate at indicated time points.

### Adipogenic differentiation *in vitro*

HucMSC and GC-MSC cells were seeded at the density of 5 × 10^5^ cells/well in 6-well plates and incubated at 37°C in 5% CO_2_ for 24 h, then treated with or without 100 or 200 ng/ml human rIL-17B for 48h. Then changed to adipogenic differentiation medium (HUXUC-90031, CyagenBiosciences, CA, USA) according to the manufacturer's instructions. At the end of induction, adipogenic potential was analyzed by oil red O staining.

### Statistical analysis

All data were shown as means ± standard deviation (SD). The statistically significant differences between groups were assessed by *t*-test using Prism software (GraphPad, San Diego, USA). *P* value < 0.05 was considered significant.
